# Pretreatment C-reactive protein to albumin ratio for predicting overall survival in advanced pancreatic cancer patients

**DOI:** 10.1038/s41598-017-03153-6

**Published:** 2017-06-07

**Authors:** Junjie Hang, Peng Xue, Haiyan Yang, Shaobo Li, Donghui Chen, Lifei Zhu, Weiyi Huang, Shujuan Ren, Yue Zhu, Liwei Wang

**Affiliations:** 1 Department of Medical Oncology and Pancreatic Cancer Center, Shanghai General Hospital, Shanghai Jiao Tong University School of Medicine, New Songjiang Road 650, Shanghai, 201620 China; 2 Shanghai Key laboratory of Pancreatic Disease, Shanghai General Hospital, Shanghai, 201620 China; 3 Department of Oncology, Renji Hospital, Shanghai Jiao Tong University School of Medicine, Pujian Road 160, Shanghai, 200120 China; 4 Pathology Center, Shanghai General Hospital, Shanghai Jiao Tong University School of Medicine, Haining Road 100, Shanghai, 200080 China

## Abstract

Although previous studies demonstrated that elevated C-reactive protein to albumin ratio (CAR) predicted poor prognosis in various solid tumors, little was known about the prognostic value of CAR in patients with advanced pancreatic cancer (APC). The aim of the present study was to assess CAR as one independent prognostic factor in predicting overall survival (OS) in APC patients who had received palliative chemotherapy. Data of 142 APC patients who received palliative chemotherapy between 2009 and 2014 were retrospectively documented. We classified the patients into two groups based on the optimal cutoff value of CAR identified by generating receiver operating characteristics (ROC) curve. The clinicopathological parameters were compared between two CAR groups. Pearson correlation test showed that the level of C-reactive protein (CRP) was inversely correlated with albumin (r = −0.387; P < 0.001). Kaplan-Meier analysis demonstrated overall survival (OS) was significantly longer in CAR < 0.156 group than CAR ≥ 0.156 group (11.2 vs 5.9 months, P < 0.001). CAR was an independent prognostic factor for OS in the Cox regression model (HR, 1.623; 95% CI, 1.093–2.410; P = 0.016). Furthermore, the discrimination ability of CAR (AUC = 0.648, P = 0.025) was slightly higher than that of other inflammation-based factors. Therefore, pretreatment CAR could be an independent prognostic biomarker for APC patients.

## Introduction

Pancreatic cancer is the seventh leading cause of cancer-related mortality among both men and women globally. In more developed regions, the incidence rate of pancreatic cancer is 8.6 per 100,000 in males and 5.9 per 100,000 in females^1^. Even with curative resection, the 5-year overall survival rate is less than 5%^2^. Most patients with locally advanced or metastatic disease at the first diagnosis can only receive the palliative chemotherapy^3^. The prognosis of advanced pancreatic cancer (APC) remains unsatisfactory.

Emerging evidence suggests the cancer-associated inflammation and nutritional status play a critical role in the progress of tumors^4^. Accordingly, previous studies identified several immunologically or nutritionally relevant biomarkers as prognostic factors for survival, such as CRP^5–7^, Glasgow prognostic score (GPS)^8^, modified Glasgow prognostic score (mGPS)^9^, neutrophil-to-lymphocyte ratio (NLR)^10^ and platelet-to-lymphocyte ratio (PLR)^11^. Among these, both GPS and mGPS are determined based on the serum concentration of CRP and albumin. As they are qualitative scores in nature, they may have the potential to cause underestimation (a lower CRP level) or overestimation (a lower albumin level) of the prognostic evaluation in cancer patients^12^.

Recently, a new prognostic index, CAR, has been reported as an independent prognostic factor in various tumors including pancreatic cancer^12–18^. Although CAR is also calculated based on the serum levels of CRP and albumin, it is a more quantitative parameter when compared with GPS or mGPS. In previous cohort study of the prognostic potential of CAR in pancreatic cancer, a large number of patients with resectable pancreatic cancer were enrolled^18^. Nevertheless, the prognostic value of CAR in APC patients who can only receive palliative chemotherapy has not been verified. Therefore, this study investigated CAR as an independent prognostic factor for overall survival (OS) in APC patients.

## Methods

### Patients

From 2009 to 2014, 142 patients with locally advanced or metastatic pancreatic cancer (ICD, Tenth Revision, codes C25) were enrolled at the Department of Oncology and Pancreatic Cancer Center, Shanghai General Hospital, Shanghai Jiao Tong University (Shanghai, China). The following inclusion criteria were applied: (1) without any concurrent cancer at another organ site; (2) with at least two cycles of palliative chemotherapy after the first diagnosis; (3) without any incomplete records of clinicopathological features; (4) pathologically confirmed pancreatic ductal adenocarcinoma. Baseline clinicopathological characteristics were retrieved from electronic medical charts and summarized in Table 1. In 101 patients with metastatic pancreatic cancer, 71 of them had liver metastasis and 30 of them had metastasis in other organs like lung, kidney and spleen. The CAR was calculated by dividing the serum CRP by the albumin obtained at the time of diagnosis. The GPS was determined as follows: the patients with a high CRP level (>10 mg/L) and a low albumin level (<35 g/L) were scored 2, those with either abnormality were given a score of 1 and those without any abnormal values were given a score of 0^19^. Likewise, the mGPS is almost the same as that of GPS except that the patients with only a low albumin level were scored 0. Palliative chemotherapy regimens included gemcitabine monotherapy (n = 50)^20^, gemcitabine combination therapy (n = 45, including gemcitabine and oxaliplatin combination therapy^21^, gemcitabine and S-1 combination therapy^22^, gemcitabine and erlotinib combination therapy^23^, gemcitabine and nab-paclitexal combination therapy^24^) and gemcitabine exclusive therapy (n = 47, including S-1 monotherapy^25^, nab-paclitexal monotherapy^26^ and FOLFIRINOX^27^). The average treatment cycles of first-line chemotherapy were 3.3. Informed consent was obtained from all subjects and all experimental protocols were approved by the Ethics Committees of Shanghai General Hospital. And the methods were carried out in accordance with the relevant guidelines and regulations.

**Table 1:** Baseline clinicopathological characteristics of patients with APC.

Valuables	Category	Characteristics
Gender	Male	92 (64.8%)
	Female	50 (35.2%)
Age	Median (Range)	61 (34–86)
ECOG PS	0	14 (9.9%)
	1	108 (76.1%)
	2	20 (14.1%)
Primary tumor location	Head and neck	61 (43.0%)
	Body and tail	81 (57.0%)
TNM stage	III	41 (28.9%)
	IV	101 (71.1%)
Liver metastasis	Yes	71 (50.0%)
	No	71 (50.0%)
Chemotherapy	Gemcitabine monotherapy	50 (35.2%)
	Gemcitabine combination therapy	45 (31.7%)
	Gemcitabine exclusive therapy	47 (33.1%)
Albumin (g/L)	Median (Range)	39.2 (26.1–48.4)
CRP (mg/L)	Median (Range)	3.55 (0.2–178.0)
CAR	Median (Range)	0.099 (0.004–5.266)
GPS	0	79 (55.6%)
	1	47 (33.1%)
	2	16 (11.3%)
mGPS	0	92 (64.8%)
	1	34 (23.9%)
	2	16 (11.3%)
AST (IU/L)	Median (Range)	25.0 (7.3–1529.0)
ALT (IU/L)	Median (Range)	20.9 (5.0–1300.0)
CA19–9 (U/ml)	Median (Range)	430.45 (0.60–2084.00)
CEA (ng/ml)	Median (Range)	6.57 (0.40–1065.00)
Hemoglobin (g/L)	Median (Range)	122 (75–168)

### Cutoff values for CAR and other factors

**Figure 1:**
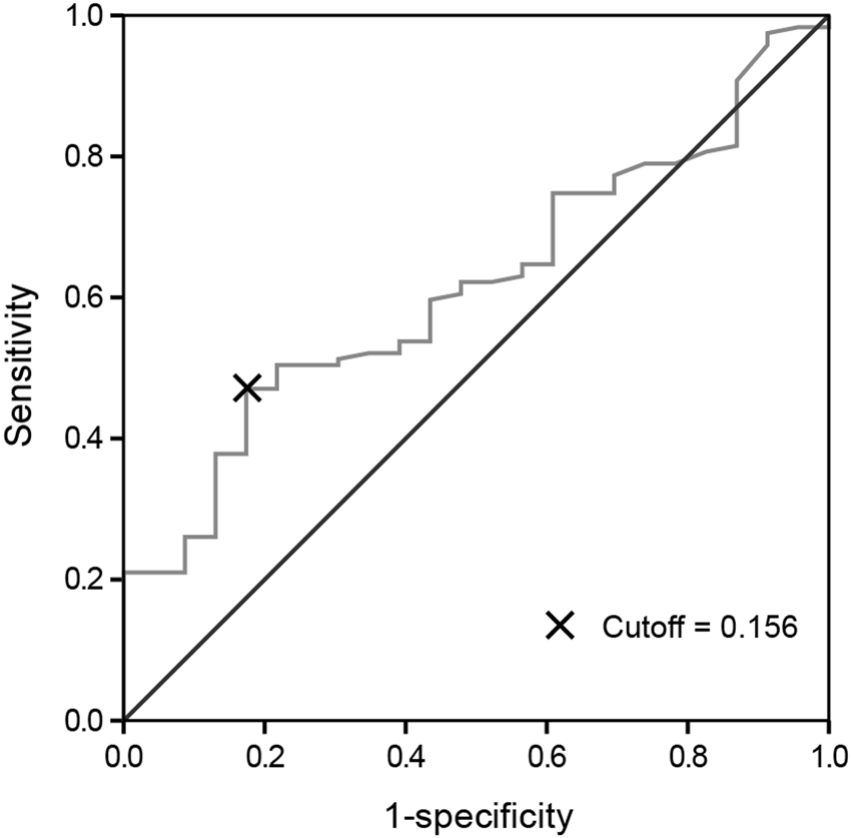
Cutoff value of CAR assessed by ROC curve.

There was no consistent cutoff value of CAR^18, 28^, thus it was identified by generating receiver operating characteristics (ROC) curve. The area under the curve (AUC) was calculated as 0.62 (95% CI, 0.51–0.73) for the CAR (Fig. 1). The CAR of 0.156 corresponded to the maximum sum of sensitivity and specificity on the ROC curve, which was equivalent to the maximization of Youden’s J statistics (J = sensitivity + specificity-1)^29^. For other factors, the cutoff values were their upper limit of normal values (AST, ALT and CEA) or those applied in other large trails (CA19–9 and hemoglobin) which were close to the median values of these factors^30^.

### Statistical analysis

All statistical analyses were performed with SPSS statistical software (version 21.0, SPSS Inc., Chicago, IL, USA). Descriptive statistics were presented as median and 95% confidence interval (95% CI). For the assessment of correlation between CAR and other valuables, patients were stratified into two groups according to different factors including gender (male and female), age (≥60 or <60 years), ECOG PS (0, 1 or 2), TNM stage (III or IV), liver metastasis (Yes or No), primary tumor location (head and neck or body and tail), chemotherapy (gemcitabine monotherapy or other therapies), CAR (≥0.156 or <0.156), Aspartate transaminase (AST) (≥40 IU/L or <40 IU/L), Alanine transaminase (ALT) (≥40 IU/L or <40 IU/L), Carbohydrate antigen 19–9 (CA19-9) (≥1000 U/ml or <1000 U/ml), Carcinoembryonic antigen (CEA) (≥5 ng/ml or <5 ng/ml) and hemoglobin (≥100 g/L or <100 g/L)^31^. Comparison between these groups was conducted using the Pearson Chi-Square test and Continuity Correction. The correlation between CRP and albumin was assessed by Pearson correlation test. OS was defined from the date of chemotherapy initiation to the date of death for any reason or censored to the last follow-up visit censored. Furthermore, survival analysis was performed with the Kaplan-Meier method and the log-rank test. Cox regression analysis was used to investigate prognostic factors for OS. By conducting ROC curve, we evaluated the specificity and sensitivity of CAR, CRP, GPS and mGPS. For each factor, we calculated the HRs and corresponding 95% CIs. Two-sided P < 0.05 was considered statistically significant.

## Results

### Patient characteristics

The baseline clinicopathological characteristics of patients with APC were summarized in Table 1. 82 patients had a pretreatment CAR of <0.156 while 60 patients had a pretreatment CAR of >0.156. We compared the clinicopathological characteristics between the two groups (Table 2). The percentages of patients with TNM stage IV, liver metastasis and AST ≥ 40 IU/L were significantly higher within the CAR ≥ 0.156 group (P < 0.05). However, percentages of patients with other variables were comparable between the two CAR groups.

**Table 2:** Baseline clinicopathological characteristics according to CAR.

Characteristics	CAR < 0.156 n = 82	CAR ≥ 0.156 n = 60	P-value
Gender			
Male	49 (53.3%)	43 (46.7%)	0.142
Female	33 (66.0%)	17 (34.0%)	
Age			
<60	39 (63.9%)	22 (36.1%)	0.195
≥60	43 (53.1%)	38 (46.9%)	
ECOG PS			
2	9 (45.0%)	11 (55.0%)	0.213
0–1	73 (59.8%)	49 (40.2%)	
Primary tumor location			
Head and neck	35 (57.4%)	26 (42.6%)	0.938
Body and tail	47 (58.0%)	34 (42.0%)	
TNM stage			
III	33 (80.5%)	8 (19.5%)	<0.001
IV	49 (48.5%)	52 (51.5%)	
Liver metastasis			
Yes	35 (49.3%)	36 (50.7%)	0.041
No	47 (66.2%)	24 (33.8%)	
Chemotherapy			
Gemcitabine monotherapy	31 (62.0%)	19 (38.0%)	0.449
Others	51 (55.4%)	41 (44.6%)	
AST (IU/L)			
<40	60 (63.8%)	34 (36.2%)	0.040
≥40	22 (45.8%)	26 (54.2%)	
ALT (IU/L)			
<40	63(59.4%)	43 (40.6%)	0.485
≥40	19 (52.8%)	17 (47.2%)	
CA19-9 (U/ml)			
<1000	51 (60.0%)	34 (40.0%)	0.507
≥1000	31 (54.4%)	26 (45.6%)	
CEA (ng/ml)			
<5	36 (64.3%)	20 (35.7%)	0.203
≥5	46 (53.5%)	40 (46.5%)	
Hemoglobin (g/L)			
<120	33 (51.6%)	31 (48.4%)	0.177
≥120	49 (62.8%)	60 (42.3%)	

### Comparison of OS stratified by pretreatment albumin, CRP and CAR

**Figure 2:**
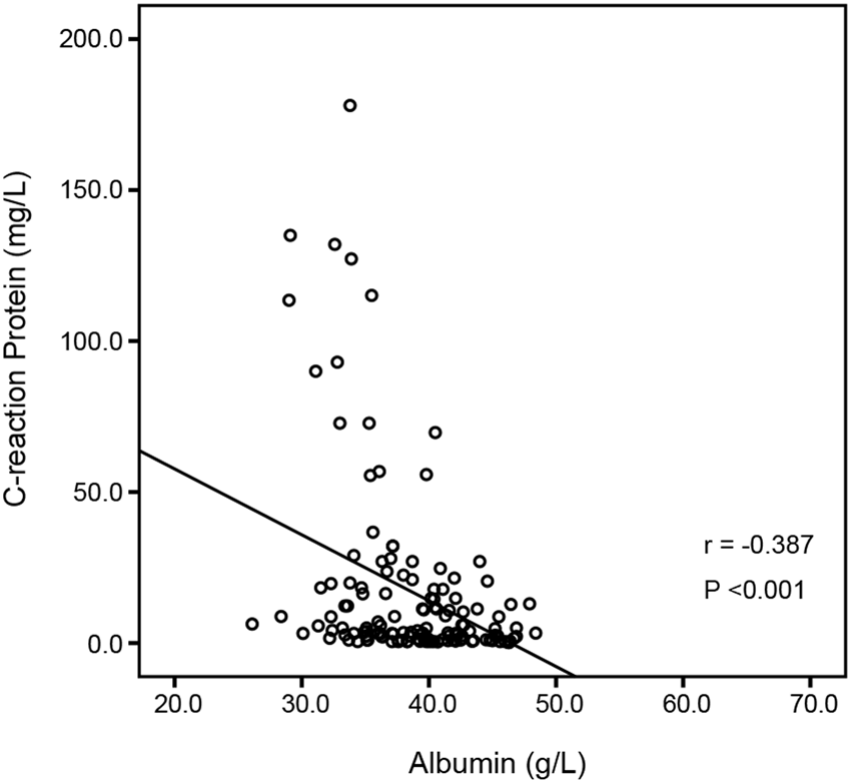
The correlation between CRP and albumin.

**Figure 3:**
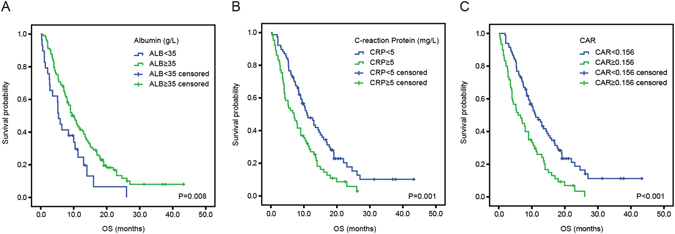
Kaplan-Meier estimates of overall survival according to the level of serum albumin (**A**), CRP (**B**) and CAR (**C**).

Pearson correlation test demonstrated that the level of CRP was inversely correlated with the level of albumin (r = −0.387; P < 0.001, Fig. 2). In the the Kaplan-Meier analysis, the median OS of patients with albumin < 35 g/L was 5.4 (95% CI: 4.3–6.5) months which was significantly shorter than 10.0 (95% CI: 8.1–11.9) months of patients with albumin ≥35 g/L (P = 0.008, Fig. 3A). Likewise, patients with CRP ≥ 5 mg/L have a poorer OS compared to those with CRP < 5 mg/L (7.0 months vs. 11.0 months, P = 0.001, Fig. 3B). Moreover, the median OS was 11.2 (95% CI: 8.5–13.9) months in CAR < 0.156 group and 5.9 (95% CI:3.0–8.8) months in CAR ≥ 0.156 group (hazard ratio (HR) 2.004, 95% CI: 1.389–2.891; P < 0.001, Fig. 3C).

### Prognostic factors for OS

In univariate analysis, five variables of ECOG PS (P = 0.005), TNM stage (P < 0.001), CAR (P < 0.001), AST (P = 0.024) and CA19-9 (P < 0.001) correlated with OS were identified. All these factors were subsequently analyzed in multivariate analysis. Consequently, TNM stage (P = 0.015), CAR (P = 0.016) and CA19- 9 (P = 0.001) were found to be independent prognostic factors (Table 3).

**Table 3:** Univariate and multivariate analysis of poor prognostic factors for OS in APC patients.

Characteristics	Univariate analysis			Multivariate analysis		
	HR	95% CI	P-value	HR	95% CI	P-value
Gender						
Male	0.988	0.673–1.452	0.952			
Female						
Age						
<60	0.876	0.609–1.259	0.475			
≥60						
ECOG PS						
2	2.011	1.233–3.280	0.005	1.524	0.886–2.261	0.128
0–1						
Primary tumor location						
Head and neck	1.375	0.948–1.996	0.093			
Body and tail						
TNM stage						
IV	2.163	1.415–3.307	<0.001	1.762	1.121–2.771	0.014
III						
Liver metastasis						
Yes	1.999	1.382–2.891	< 0.001			
No						
Chemotherapy						
Gemcitabine monotherapy	0.831	0.573–1.207	0.331			
Others						
CRP (mg/L)						
≥5	1.793	1.245–2.580	0.002			
<5						
Albumin (g/L)						
≥35	0.553	0.354–0.866	0.010			
<35						
CAR						
≥0.156	2.004	1.389–2.891	<0.001	1.629	1.097–2.419	0.016
<0.156						
GPS						
2	1.539	1.201–1.971	0.001			
1						
0						
mGPS						
2	1.437	1.121–1.844	0.004			
1						
0						
AST (IU/L)						
≥40	1.560	1.059–2.297	0.024	0.937	0.604–1.453	0.771
<40						
ALT (IU/L)						
≥40	1.087	0.713–1.658	0.697			
<40						
CA19–9 (U/ml)						
≥1000	1.989	1.359–2.911	<0.001	1.973	1.332–2.924	0.001
<1000						
CEA (ng/ml)						
≥5	1.380	0.948–2.010	0.092			
<5						
Hemoglobin (g/L)						
<120	0.887	0.618–1.274	0.516			
≥120						

### Subgroup analysis and discrimination ability of CAR

**Figure 4:**
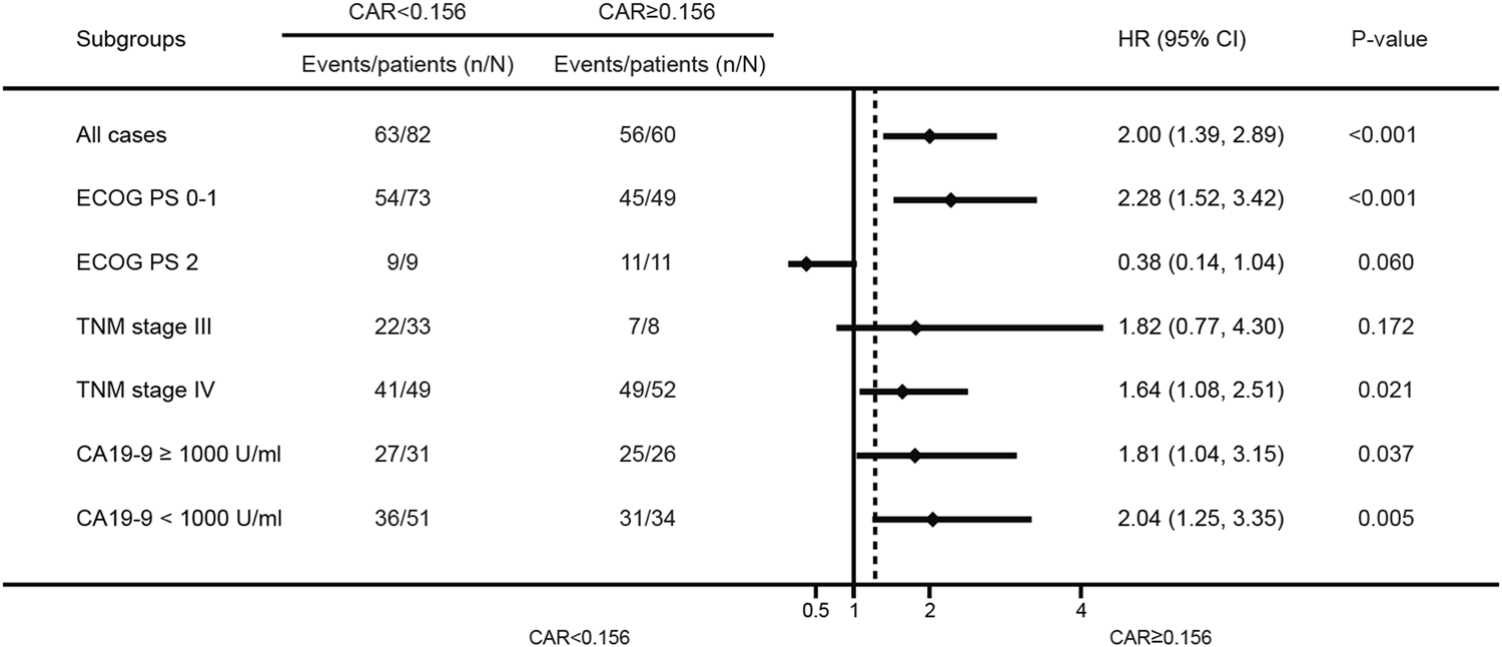
Hazard ratios (HRs) of CAR in different patient subgroups identified by ECOG PS, TNM stage and CA19-9. HRs >1.0 indicate a worse outcome.

CAR was significantly correlated with OS in the subgroup identified by CA19-9. However, CAR demonstrated no correlation with OS in the subgroup of patients with ECOG PS 2 or TNM stage III (Fig. 4).

**Figure 5:**
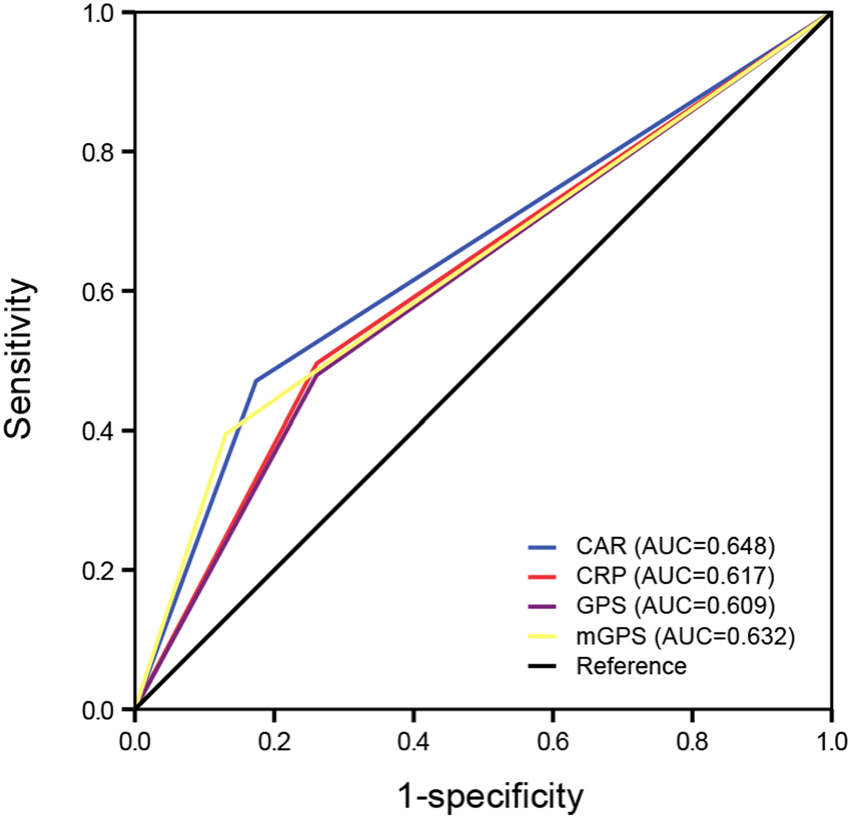
The predictive ability of the four inflammation-based prognostic scores was compared by ROC curves.

ROC curves were used to evaluate the discrimination ability of CAR and other inflammation-based factors including CRP, GPS and mGPS (Fig. 5). The discrimination ability of CAR, as assessed by AUC, was 0.648 (P = 0.025), which was the highest among these inflammation-based factors (CRP 0.617, GPS 0.615, and mGPS 0.632).

## Discussion

In the present study, pretreatment CAR was found to be an independent prognostic factor for overall survival in APC patients receiving palliative chemotherapy. Haruki, et.al showed that elevated pretreatment CAR predicted poor clinical outcomes in pancreatic cancer patients with resectable tumors in 2016^18^. More recently, Mengwan Wu, et.al investigated the prognostic value of CAR in pancreatic cancer patients treated with or without chemotherapy^28^. However, there was optimal difference in the cutoff values of CAR identified in these two study, which could be explained by the different populations of patients enrolled in two studies.To the best of our knowledge, this is the first study to evaluate the prognostic value of CAR in a cohort of APC patients receiving palliative chemotherapy.

Systemic inflammation response plays a vital role in the progression of pancreatic cancer. Various prognostics scoring models assessed by peripheral blood cell count or inflammatory factors were developed retrospectively to stratify the optimal pancreatic cancer patients receiving palliative care^32^. However, little has been used predicatively in clinical practice.

CRP, a marker of inflammation, was correlated with survival outcomes in various cancers, including pancreatic cancer^6, 7, 33^. On the other hand, hypoalbuminemia, an indicator for chronic malnutrition, is also a common complication for advanced cancer patients. Therefore, the CAR, a combined pattern of both CRP and albumin, may reveal the outcome of pancreatic cancer in a better way. Haruki, et.al found that patients in high CAR group happened to be in more advanced TNM stage (p = 0.007). Such finding was consistent with this study as the percentages of patients with TNM stage IV, liver metastasis and AST ≥ 40 IU/L were significantly higher within the CAR ≥ 0.156 group than CAR < 0.156 group (P < 0.05), which may have reflected the poorer status of patients with this disease. However, after adjustment for TNM stage, AST, ECOG PS and CA19-9 in multivariate analysis, the CAR < 0.156 remained favorable independent of prognostic factor, with a clinically relevant HR value (HR 1.629, 95% CI 1.097–2.419; P = 0.016), which suggested the different prognosis of CAR stratification was not merely attribute to the difference in baseline characteristics between the two groups. Furthermore, the subgroup analysis of CAR in patients with TNM stage IV also demonstrated the prognostic value of CAR regardless of TNM stage (HR: 1.64, 95% CI 1.08–2.51; P = 0.021). Our study also showed there was a reciprocal relationship between CRP and albumin (r = −0.387, P < 0.001, Fig. 2). This is consistent with Hwang JC’s work^34^ and can be partly explained by the reason that inflammation reduces albumin concentration by decreasing its synthesis rate^35^. In addition, immunonutrition can also suppress the inflammatory response^36^.

Previous studies revealed that GPS or mGPS could be independent prognostic factors in pancreatic cancer patients^37–39^. However, in this study, CAR showed superior discrimination ability than other inflammation-based scores including GPS and mGPS in pancreatic cancer patients, which was consistent with the results of several studies conducted among patients with other cancers types^12, 14^. Furthermore, Haruki, et.al also found CAR (P = 0.035), rather than mGPS (P = 0.091), was independent and significant predictor of the OS. This may be partially explained by the reason that CAR is a simple ratio with a continuous range of values but both GPS and mGPS, consisting of dichotomized variables, have a qualitative nature with discontinuous values.

The subgroup analysis (Fig. 4) showed that the prognostic value of CAR in high CA19-9 or low CA19-9 patients were also identified respectively. This means that the CAR with cutoff value of 0.156 may also stratify high or low CA19-9 patients into two groups with prominent difference in OS.

There are several strengths of this study. First, this study boasts a cohort with long follow-up period. Second, CAR is a biomarker that can be utilized in clinical practice as the measurement of CAR is non-invasive, easy to acquire and affordable for the patients. Several limitations of this study should also be acknowledged. One potential limitation is that it is a retrospective and single-center study with relatively small sample size which may cause selection bias. Second, this study mainly focused on the pretreatment CAR which may be largely affected by other factors like infection or cancer complication. Third, heterogeneous treatments in this study may affect survival outcome although we found chemotherapy was not correlated with OS in this study as some other studies had reported^10, 40^. Both CRP and albumin are produced in liver and various chemotherapy regimens have different effects on patients’ liver function and inflammation status, which may affect the production of CRP and albumin. Another limitation is the lack of a validation cohort to confirm the cutoff and prognostic value of CAR. Therefore, future study on a larger sample size and same treatment modality should be conducted to verify the findings in this study. Finally, the concrete mechanisms underlying the prognostic value of CAR should be further investigated.

In conclusion, this study indicates that the pretreatment CAR could be an independent prognostic biomarker for APC patients.
